# Influence of an Acute Exposure to a Moderate Real Altitude on Motoneuron Pool Excitability and Jumping Performance

**DOI:** 10.3389/fphys.2022.861927

**Published:** 2022-04-25

**Authors:** Igor Štirn, Amador Garcia-Ramos, Belen Feriche, Vojko Strojnik, Katja Tomažin

**Affiliations:** ^1^ Faculty of Sport, University of Ljubljana, Ljubljana, Slovenia; ^2^ Department of Physical Education and Sport, Faculty of Sport Sciences, University of Granada, Granada, Spain; ^3^ Department of Sports Sciences and Physical Conditioning, Faculty of Education, Universidad Catolica de la Santisima Concepcion, Concepción, Chile

**Keywords:** drop jump, H-reflex, hypoxia, squat jump, altitude

## Abstract

The aim of the study was to test whether ascending to a moderate real altitude affects motoneuron pool excitability at rest, as expressed by a change in the H-reflex amplitude, and also to elucidate whether a possible alteration in the motoneuron pool excitability could be reflected in the execution of lower-body concentric explosive (squat jump; SJ) and fast eccentric-concentric (drop jump; DJ) muscle actions. Fifteen participants performed four experimental sessions that consisted of the combination of two real altitude conditions [low altitude (low altitude, 690 m), high altitude (higher altitude, 2,320 m)] and two testing procedures (H-reflex and vertical jumps). Participants were tested on each testing day at 8, 11, 14 and 17 h. The only significant difference (*p* < 0.05) detected for the H-reflex was the higher H-reflex response (25.6%) obtained 15 min after arrival at altitude compared to baseline measurement. In terms of motor behavior, DJ height was the only variable that showed a significant interaction between altitude conditions (LA and HA) and time of measurement (8, 11, 14 and 17 h) as DJ height increased more during successive measurements at HA compared to LA. The only significant difference between the LA and HA conditions was observed for DJ height at 17 h which was higher for the HA condition (*p* = 0.04, ES = 0.41). Although an increased H-reflex response was detected after a brief (15–20 min) exposure to real altitude, the effect on motorneuron pool excitability could not be confirmed since no significant changes in the H-reflex were detected when comparing LA and HA. On the other hand, the positive effect of altitude on DJ performance was accentuated after 6 h of exposure.

## Introduction

Acute and chronic exposures to altitude can affect both the physiological responses and the locomotor behavior of the human body (Mazzeo, 2008; West, 2017; Dietz and Hackett, 2019) The main effect of the ascent to altitude is a progressive reduction in the partial pressure of O_2_ in the inspired air. It is well known that the reduced availability of O_2_ is responsible for the reduction in performance during tasks where the predominant source of energy for muscle contraction is derived from the aerobic system (e.g., long-distance running) (Barnes and Kilding, 2015; Sinex and Chapman, 2015; Flaherty et al., 2016; Sharma et al., 2018; Mujika et al., 2019). Although acute exposure to altitude impairs endurance performance, chronic exposure to hypoxia can induce a number of cardiovascular and hematological adaptations that are potentially effective to enhance performance in endurance tasks (Steele et al., 2012; Wyatt, 2014; Burtscher et al., 2018). Therefore, it is not surprising that altitude training is a common preparation strategy for many elite and high-level athletes specialized in endurance sports (e.g., runners, swimmers, or cyclists). It is noteworthy that moderate altitude (1,500–3,500 m) is the condition most commonly used by athletes for their altitude training camps (Dietz and Hackett, 2019). In addition, there are a number of strategies for conducting altitude training camps, the most common of which are the following: (I) Live and train at altitude, (II) live at altitude and train at sea level, (III) live at altitude and train at both altitude and sea level, and (IV) live at sea level and train at altitude (Sinex and Chapman, 2015). Depending on the desired physiological adaptations and considering logistical constraints, athletes can choose the altitude training strategy that best fits their goals.

In contrast to endurance performance, there is some scientific evidence indicating that acute exposure to moderate altitude may actually improve performance in tasks where maximal strength and power capacities are of paramount importance (e.g., unloaded and loaded vertical jumps) (Feriche et al., 2014; García-Ramos et al., 2016; Feriche et al., 2017). One of the potential mechanisms responsible for these findings could be an increase in the motoneuron pool excitability. Higher excitability of the motoneuron pool positively affects performance during explosive actions and may allow for a more pronounced reflex response during tasks that involve a fast stretch-shortening cycle (SSC) (Komi and Gollhofer, 1997; Markovic and Mikulic, 2010). Two of the vertical jumps most commonly used to test lower body ballistic performance are the squat jump (SJ) and drop jump (DJ). The SJ demonstrates the ability of the muscles to produce force in a purely concentric manner, whereas the DJ consists of a fast SSC involving a reflex response (Zuur et al., 2010). Therefore, a practical approach to test the hypothesis that acute altitude exposure increases the excitability of the motoneuron pool would be to examine changes in jump performance at altitude.

Motoneuron excitability can be observed at multiple sites along the motor pathway, and various neural assessment tools have been used to monitor the direct and indirect effects of hypoxia on motor behavior. Cortical stimuli using transcranial magnetic stimulation (Szubski et al., 2006) and subcortical stimuli using cervicomedullary electrical simulation (Ruggiero et al., 2018) have been used to examine the responsiveness of the motor neuron pool, provided that the excitability of the peripheral nerves is taken into account. Electromyography can also be used to record the amplitude of the EMG signal and M, F, and H waves. The latter (H-reflex) is one of the commonly used tests to determine the motoneurons excitability (Palmieri et al., 2004; McNeil et al., 2013). The H-reflex amplitude depends directly on the motoneuron excitability and the level of presynaptic inhibition exerted on Ia afferents (McNeil et al., 2013). Of note is that presynaptic inhibition has been reported to be comparable at rest and during a steady contraction (Meunier and Pierrot-Deseilligny, 1989; Nielsen and Kagamihara, 1993).

Most studies that addressed the changes in H-reflex under hypoxia were performed under normobaric conditions with varying hypoxic dose and duration. It has been shown that the excitability of motor neurons in acute normobaric hypoxia at rest is not different from that in normoxia (Szubski et al., 2006; Goodall et al., 2010; Millet et al., 2012; Rupp et al., 2012; Goodall et al., 2014). On the other hand, Rupp, et al. (2015) measured the H-reflex before and after a sustained contraction of plantar flexors at 40% of maximal voluntary isometric contraction (MVC) under two conditions differing in the inspired fraction of O2 (FiO2 = 0.21 and 0.11 at normoxia and hypoxia, respectively). They reported that the ratio between maximum amplitudes of H-reflex and M-wave (H/M ratio) measured before exercise was depressed at hypoxia. However, they later found that it recovered significantly (still under hypoxia) during the recovery phase, whereas there was no recovery under normoxia. Delliaux and Jammes (2006) showed that an acute exposure to hypoxia (participants inhaled a gas mixture containing 15% of O_2_ and 85% of nitrogen) increased the amplitude of the H-reflex, which started 10 min after hypoxia exposure and persisted during the first 20 min of recovery. They suggested that the facilitation of the H-reflex might be due to the direct effects of hypoxemia on the supraspinal structures controlling the H-reflex or to the activation of the of the III and IV afferents. Indeed, some observations suggest that stimulation of the III and IV afferents in cats may facilitate muscle contraction by the reinforcement of the fusimotor neuron-muscle spindle-motoneuron path (Jovanovic´ et al., 1990; Ljubisavljevic´ et al., 1995). If we assume that acute exposure to real altitude produce such facilitation, mechanically induced stretch reflex during DJ should be pronounced and concomitantly DJ height would be increased. In addition, Willer et al. (1987), also observed a 50% increase in the H-reflex amplitude after an exposure of 12 min to hypoxia. Based on their results, they proposed that the effects of hypoxia on the nervous system consist of a direct depolarizing effect of peripheral *α*-fibers and sensory Ia-fibers and a central effect on supraspinal structures affecting spinal *α*-motoneurons.

Although physical responses to exercise in normobaric hypoxia have been extensively studied, it is not yet clear how the exposure to hypobaric hypoxia affects anaerobic performance. At this point we should emphasize that normobaric hypoxia is not a surrogate for hypobaric hypoxia and cannot be used interchangeably (Fulco et al., 2011; Millet and Debevec, 2020). Therefore, changes in spinal excitability should also be monitored at real altitude. As far as we know, there are only two studies that have measured the H-reflex at real altitude. Kayser et al. (1993) studied the H-reflex after a 1-week acclimatization period between 2,850 and 5,050 m and reported that the H/M ratio remained unchanged. On the contrary, Schmeling et al. (1977) reported a decrease in the amplitude of the H-reflex 6 h after ascent to 3,200 m, which was followed by an increase in the amplitude after the acclimatization period of 5–14 days. Both studies were conducted at a very high real altitude, which is not normally used by athletes in the live-low train-high strategy. In addition, the changes in the H-reflex obtained at real altitude, were observed under chronic conditions (>6 h exposure), whereas the live-low train-high strategy usually uses a shorter hypoxic exposure (<6 h).

Therefore, the primary aim of our study was to test whether ascending to a moderate real altitude affects motoneuron pool excitability at rest, as expressed by a change in the H-reflex amplitude. Furthermore, we also aimed to elucidate whether a possible alteration in the motoneuron pool excitability could be reflected in a change in motor behavior assessed through the execution of lower-body concentric explosive (SJ) and fast eccentric-concentric (DJ) muscle actions. We hypothesized that acute exposure to a moderate real altitude would increase the H-reflex response, which would be associated with an improvement in jump performance. This improvement would be more accentuated for DJ compared to SJ.

## Materials and Methods

### Participants

Fifteen sport science students, three females and twelve males, volunteered to participate in the study (age: 21.9 ± 2.3 years, body mass: 69.6 ± 7.7 kg, stature: 177.1 ± 4.8 cm). They were all healthy and physically active. All participants were introduced to the aims and protocols of the study before signing a written consent form. The study protocol adhered to the tenets of the Declaration of Helsinki and was approved by the institutional review board of the University of Granada. Participants were informed of possible symptoms of acute altitude sickness and carefully monitored throughout the protocol for dizziness, headache, sleepiness and nausea.

### Study Design

A repeated-measures design was used with participants attending to the laboratory on five separate days, which were separated 72 h to avoid resistance priming effect (Harrison et al., 2019). The first session was used for familiarization purposes in which the participants were introduced to the M-wave and H-reflex measurement protocol and demonstrated a proper execution technique during the squat jump (SJ) and drop jump (DJ). The four remaining experimental days were carried out in a randomized and counterbalanced order. The four sessions consisted of the combination of two terrestrial altitude conditions [low altitude (LA): 690 m asl at the Faculty of Sport Sciences of the University of Granada; higher altitude (HA): 2,320 m asl (moderate altitude) at the High Performance Centre of Sierra Nevada, partial pressure for inspired O2 112.2 mmHg (Gallagher and Hackett, 2004)] and two testing procedures [H-reflex and vertical jumps (SJ and DJ) measurements]. Participants were tested at four time points during each testing day (8:00, 11:00, 14:00, and 17:00 h). The timeline resembles the duration of one or two training sessions per day, as is common in the live-low-train-high strategy. It was shown that corticospinal excitability increased after 3 h and was unaffected after 1 h (Rupp et al., 2012).

Of note is that the first measurement of all testing days at 8:00 h was always performed at LA to check the repeatability of the measurements. For the HA condition, the same researcher was responsible for driving the participants by car at normal speed after the 8:00 h measurement. The trip started at 09:50 h and lasted approximately 45 min. The first measurement at HA was performed 10–20 min after the arrival to HA. During the testing days, we asked participants not to be physically active between measurements to avoid potential effects of muscle fatigue or potentiation, and they were also instructed to avoid strenuous exercises the day before testing. Participants remained in the Faculty of Sport Sciences or in the High Performance Centre of Sierra Nevada between the successive measurements of the same testing day.

### H-Reflex Measurements

No warm up was performed before H-reflex measurements. H-reflexes were elicited from the soleus muscle in supine position after 10 min of rest, thus excluding all causes of increased excitability other than the environment (altitude). First, the optimal position for percutaneous electrical stimulation of the tibial nerve was determined with single rectangular pulses (1 ms) delivered to the right tibial nerve via a surface cathode (ø 24 mm; Kendall, Covidien, Mansfield) manually pressed into the popliteal fossa and with the anode (50 × 50 mm; Axelgaard Manufacturing, Co., LTD., Fallbrook, CA, United States) at the patella. The optimal cathode position was determined by carefully moving the electrode until an optimal response was observed in the form of a clear and distinct biphasic M-wave signal at a submaximal (lowest) stimulus intensity. Electromyography (EMG) activity of the soleus muscle was recorded with electromyographic (electrodes Kendall) placed on the one-third distal part of the length between the malleolus medialis and epicondyle medialis of the tibia, following the SENIAM recommendations. The interelectrode distance was 25 mm. A reference electrode was placed on the lateral malleolus of the right leg. The EMG signals were amplified by an octal bio amplifier (ML138; ADInstruments, Bella Vista, Australia) with a bandwidth frequency ranging from 3 to 1,000 Hz (input impedance = 200 MΩ), common mode rejection ratio = 85 dB, gain = 1,000) and analyzed using LabChart7 software (ADInstruments, Bella Vista, Australia).

To determine the M-wave and H-reflex recruitment curves, the posterial tibial nerve was stimulated with a constant current electrical stimulator (DS7A; Digitimer, Hertfordshire, United Kingdom). First, we determined the stimulation intensities for H-wave threshold and M_max_. Then, a randomized sequence was generated and manually administered to participants starting at 1 mA below the H-wave threshold to 1.5 times mA above M_max_, with gradations from 0.1 to 5 mA depending on the recruitment zone, using the smallest gradation to accurately define the H_max_ or M_max_ response. When H_max_ and M_max_ responses were visible, at least 5 stimulations were performed at that intensity. Post-activation depression during H-reflex measurements was avoided by maintaining an interval of 10–15 s between stimuli (Burke, 2016). In addition, measurements of H-reflex amplitudes and M-wave amplitudes were recorded in real time during the mapping of H-reflex and M-wave recruitment curves for each participant using custom software (Recruitment curve, Faculty of Sport, Ljubljana). More specifically, during the measurement, the amplitude of the H-reflex was plotted against the amplitude of the M-wave in real time. Subsequently, the stimulation intensity could be defined for the maximal H-reflex amplitude and M_max_. At least 5 electrical stimuli of this intensity were administered, and the amplitude of the H reflex was simultaneously monitored on the screen. Because of the possible effects of head posture on the peak-to-peak amplitude of the H reflex (Schieppati, 1987), participants were instructed to avoid head movements during the stimulation protocol. The highest peak-to-peak amplitude for the M wave (M_max_) and H reflex (H_max_) was determined from the unrectified EMG signals. However, for statistical analysis, we used the average of five EMG responses measured at the current levels used for H_max_ and M_max_. The following parameters were then calculated: 1) the peak-to-peak amplitude of the H-wave (H_max_), and 2) the peak-to-peak amplitude of the M-wave (M_max_). Then, the ratio between H_max_ and M_max_ (H/M ratio) was calculated and used for statistical analyses.

### Squat Jump and Drop Jump Measurements

Participants warmed up by stepping on a 25 cm high box for 6 min, changing the leading leg every minute, and with a pace of 30 steps per minute. Afterwards, some low intensity dynamic leg exercises were performed for 3 min, and after 2 min of rest, the jumps were performed. Each participant performed three SJs and then three DJs. There was a 30-second rest period between jumps. A force plate (9253A11, Winterthur, Switzerland) with the ARS software (S2P, Slovenia) were used for data acquisition and analysis. Only the jump that revealed the highest jump height from the three trials of the same jump modality (SJ and DJ) was used for statistical analyses.

*- Squat jump (SJ).* The participant stepped onto a force plate and stood in a half squat position defined by a 90° hip and knee flexion, with the arms on the hips, head facing straight forward. Participants were instructed to jump as high as possible without performing any countermovement.
*- Drop jump (DJ).* The participants stepped on a 25 cm high box that was placed next to the force plate. They stepped with one leg on the edge of the box and extended the other leg in the air in front of their body so that it hanged over the edge of the box above the force plate. The participant then coupled the rear leg to the front leg, jumped off the box on a force plate, and then performed a fast SSC. They were instructed to jump as high as possible with a short contact time (<220 ms), while the hips and knees were only allowed to bent slightly. The jump height and the reactive strength index [RSI = jump height (m)/contact time (s)] were used for statistical analyses.

### Statistical Analyses

Descriptive data are presented as means and standard deviations. The normal distribution of the dependent variables (SJ height, DJ height, RSI, and H/M ratio) was confirmed by the Shapiro-Wilk test (*p* > 0.05). The reliability of the dependent variables was assessed through paired samples t-tests, the Cohen’s *d* effect size (ES), the standard error of measurement expressed in relative terms through the coefficient of variation (CV), the intraclass correlation coefficient (ICC), and the corresponding 95% confidence intervals. Acceptable indices of reliability were considered to be an ICC >0.70 and a CV < 15% (Haff et al., 2015) A two-way [altitude condition (LA and HA) × time of measurement (8, 11, 14, and 17 h)] repeated measures analysis of variance (ANOVA) with Bonferoni post hoc corrections was applied on each dependent variable. The Cohen’s ES was also calculated to quantify the magnitude of the differences between the LA and HA conditions at each time point. The magnitude of the ES was interpreted as follows: trivial (<0.20), small (0.20–0.59), moderate (0.60–1.19), large (1.20–2.00), and very large (>2.00) (Hopkins et al., 2009). Reliability was assessed by means of a custom Microsoft Excel spreadsheet (Hopkins, 2000), while other statistical analyses were performed using the software package SPSS (IBM SPSS version 22.0, Chicago, IL). Alpha level of significance was set at 0.05.

## Results

**FIGURE 1:**
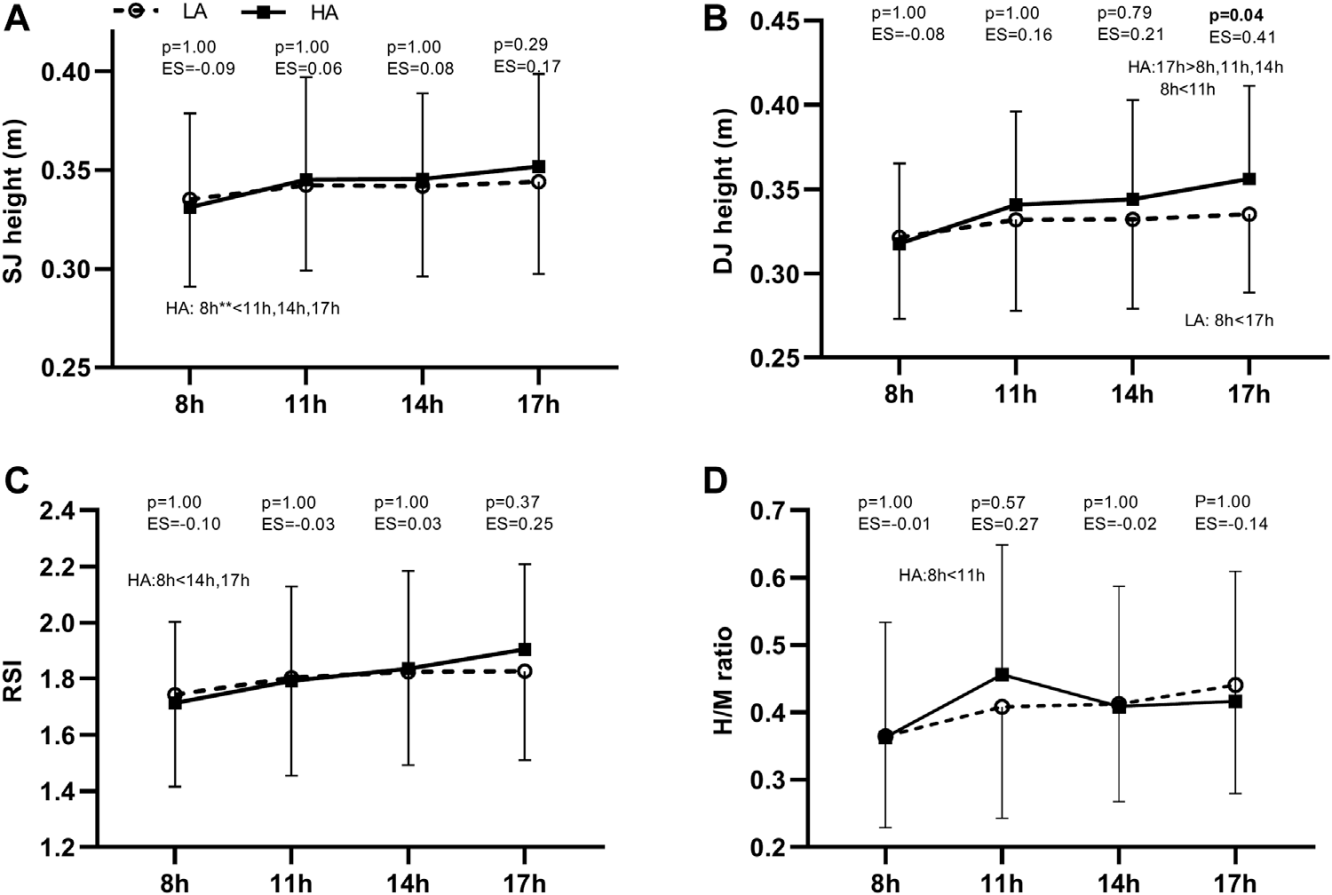
Comparison SJ height **(A)**, DJ height **(B)**, RSI **(C)**, and H/M ratio **(D)** between the low altitude (LA; open circle, dashed line) and high altitude (HA; black square, solid line) conditions at the different points of measurement. SJ, squat jump; DJ, drop jump; RSI, reactive strength index; H/M ratio, ratio between the peak-to-peak amplitude for the H-reflex and M-wave; ES, Cohen’s d effect size [(mean HA–mean LA)/SD both]; **, significant differences (p < 0.05) between the points of measurement. Data are depicted as means and standard deviations.

The four dependent variables revealed an acceptable reliability (CV < 15% and ICC >0.70) with the only exception of the H/M ratio that showed a CV of 18.5% (Table 1). The main effect of the altitude condition (Figure 1) was not significant (SJ height: F = 0.9, *p* = 0.348; DJ height: F = 2.6, *p* = 0.132; RSI: F = 0.1, *p* = 0.785; H/M ratio: F < 0.1, *p* = 0.828). On the contrary, the main effect of time always reached statistical significance (SJ height: *F* = 8.3, *p* < 0.001; DJ height: F = 20.0, *p* < 0.001; RSI: F = 12.6, *p* < 0.001; H/M ratio: *F* = 6.5, *p* = 0.001) due to the generally lower values at 8 h compared to 11, 14, and 17 h. The interaction altitude condition × time of measurement was significant for DJ height (F = 3.5, *p* = 0.022) due to the larger increments in DJ height, when compared to baseline values (i.e., 8 h), at 11, 14 and 17 h for the HA condition than for the LA condition. However, the remaining interactions were not statistically significant (SJ height: F = 1.4, *p* = 0.269; RSI: F = 1.0, *p* = 0.424; H/M ratio: F = 2.3, *p* = 0.094). The post-hoc comparisons with the magnitude of the differences are depicted in Figure 1. The only significant difference between the LA and HA conditions was observed for DJ height at 17 h which was higher for the HA condition (*p* = 0.044, ES = 0.41).

**TABLE 1:** Repeatability of the dependent variables obtained by comparing the measurements at 8 h.

Variable	Low altitude (Mean +SD)	High altitude (Mean +SD)	*p*-value	ES	CV (%) (95% CI)	ICC (95%CI)
SJ height (m)	0.335 ± 0.044	0.331 ± 0.048	0.45	−0.09	4.2 (3.1–6.6)	0.92 (0.77–0.97)
DJ height (m)	0.321 ± 0.048	0.318 ± 0.048	0.59	−0.08	5.7 (4.2–9.1)	0.87 (0.67–0.96)
RSI (m·s^−1^)	1.742 ± 0.327	1.712 ± 0.288	0.62	−0.09	9.1 (6.7–14.4)	0.77 (0.43–0.91)
Hmax (mV)	0.98 ± 0.49	0.88 ± 0.46	0.10	−0.21	12.5 (8.6–22.8)	0.95 (0.81–0.98)
Mmax (mV)	2.67 ± 0.95	2.50 ± 0.94	0.07	−0.18	6.8 (4.7–12.3)	0.97 (0.89–0.99)
H/M ratio	0.364 ± 0.136	0.363 ± 0.171	0.956	−0.01	18.5 (13.4–29.8)	0.84 (0.56–0.94)

SJ, squat jump; DJ, drop jump; RSI, reactive strength index; H/M ratio, ratio between the peak-to-peak amplitude for the H-reflex and M-wave; SD, standard deviation; ES, Cohen’s *d* effect size; CV, coefficient of variation; ICC, intraclass correlation coefficient; CI, confidence interval.

## Discussion

This study was designed to clarify whether ascending to a real moderate altitude affects the excitability of the motoneuron pool and whether this could consequently affect participants’ SJ and DJ performance. Interestingly, although a significant increase in the H/M ratio was observed 15 min after the arrival to altitude (8 vs. 11 h), no significant change was observed at 11 h between LA and HA. Therefore, we could not unequivocally confirm that environmental factors caused the observed increase in the H/M ratio. Regarding motor behavior, DJ increased slightly at HA whereas SJ remained virtually unchanged. Moreover, at 17 h (after 6 h of real altitude exposure) an improvement in DJ height was observed (0.335 ± 0.047 m vs. 0.356 ± 0.055 m, *p* = 0.04).

In order to check the repeatability of our variables and ensure similar baseline values for both altitude conditions, the first measurement of each testing day (8 h) was always performed at a low altitude. Comparison of baseline values always revealed non-significant (*p* > 0.05) and trivial differences (ES < 0.20) revealing a similar performance, while reliability was high for SJ height and DJ height and a bit lower for RSI and H/M ratio. Of note is that when we continued the measurements every 3 h at LA, no significant differences were reached between the different time points for any variable with the only exception of DJ height, which was higher at 17 h compared to 8 h. The resistance exercise priming effect or the circadian rhythm could be responsible for this significant difference (Cook et al., 2014; Harrison et al., 2019).

It is worthnoty to underline there were significant differences between the H/M ratios measured at 8 h (on HA measuring day) and at 11 h just after arrival at the altitude (*p* = 0.01). Whether these differences in the H/M ratios between 8 and 11 h at HA were due to the effect of altitude conditions remains unclear, as we found no significant differences in the H/M ratio between the HA and LA (measured on two different days) at 11 h. However, the increased excitability did not persist three and 6 h after the baseline measurement. Transient increase in H/M ratio was also observed by Delliaux and Jammes (2006), who observed that 10 min after the onset of normobaric hypoxia, the maximal amplitude of the H-reflex increased significantly and persisted for 20 min. Although their increases were larger than in our experiment (250 vs. 25.6%), the acute response of the H-reflex proceeded in the same manner and was attributed to the direct effects of hypoxemia on the supraspinal structures controlling the H reflex or to the activation of the III and IV afferents. Indeed, it has been shown that acute arterial hypoxemia increases the baseline discharge frequency of group III and especially of group IV muscle afferents in resting animals (Hill et al., 1992; Lagier-Tessonnier et al., 1993; Arbogast et al., 2000). It has been shown that discharges from III and/or IV muscle afferents reflexively increase fusimotor discharge rate (Jovanovic´ et al., 1990; Ljubisavljevic´ et al., 1995). If such facilitation of III and IV afferents occurred also in our study, this could explain transient increase in the H/M ratio as well. Assuming this was the prevailing mechanism triggered by hypoxia, then an increase in DJ height might be expected due to increased fusimotor discharge rate. Nevertheless, significant increment in DJ height was not observed until 17 h, i.e., 6 h after arrival at real altitude.

The second mechanism proposed by Delliaux and Jammes (2006), i.e., the direct effect of hypoxemia on supraspinal structures, might be functionally more important for the increase in H/M ratio at 11 h. Indeed, it has been suggested that hypoxia triggers upregulation of monoaminergic systems (Trumbower et al., 2012; Dale et al., 2014), which improve neuro-motor function of respiratory (Mitchell and Terada, 2011) and non-respiratory muscles (Trumbower et al., 2012). Specifically, it has been shown that isometric strength of plantar flexors increases after acute intermittent hypoxia following spinal cord injury (Trumbower et al., 2012). However, a similar improvement in neuro-motor function has not yet been confirmed for whole-body exercises that involve many joints and muscle groups, i.e., DJ and SJ. Therefore, a possible increase in neuro-motor function due to hypobaric hypoxia could be masked by the complex intra-muscular coordination during DJ and SJ.

Regardless of altitude, both SJ (not significantly) and DJ (significantly) increased throughout the day. Similar results were reported by Álvarez-Herms et al. (2015), who found average and peak power output during explosive strength training were not affected by moderate hypoxia, but it did slightly increase at high hypoxia. We consider at least two possible reasons: resistance exercise priming also called the “delayed potentiation effect” (Harrison et al., 2019) and circadian rhythm (Cook et al., 2014). The potential effect of exercise priming is supported by the findings of Saez de Villarreal et al. (2007) who found an increase in DJ heights 6 h after a high-intensity [up to 95% of the one-repetition maximum (1RM)] dynamic warm-up exercises (such as loaded parallel squats and countermovement jumps). In our case, the dynamic exercises that has preceded the testing set of jumps were presented by a previous testing set itself- a set of three maximal SJ and three maximal DJ. This means that testing at 8 h could have a priming effect on testing 3, 6, and 9 h later, and testing at 11 h could affect testing at 14 and 17 h, and so on. This could be supported by the review of Harrison et al. (2019) who revealed that in addition to traditional high load exercise (≥85% 1RM), low load ballistic exercise (30–40% 1RM) appeared to be the most effective and caused a delayed potentiation effect. Cook et al. (2014) also observed that performing a strength training session in the morning improved vertical jump, 40 m sprint time, and 3RM bench press and squat in the afternoon. Authors attributed their results to circadian rhythm, which may also play a role in explaining our results. Namely, the priming effect cannot accumulate endlessly, so jump heights would continuously increase after each set of test jumps. Therefore, another factor should explain the increase in DJ heights in our study. As suggested earlier, improved physical performance in the afternoon compared to the morning could be a reflection of circadian rhythms (Grgic et al., 2019).

Finally, no differences were found in the RSI with respect to altitude, except that the value at the initial measurement at 8 h for the HA was significantly lower than at 14 and 17 h. Since no significant differences were observed in DJ contact times (data not shown), this was likely due to the increased DJ height. This result is consistent with the findings of Tsoukos et al. (2018) who found an increase in RSI between 6 and 48 h after resistance priming and suggested that these improvements were likely to originate from neuromuscular changes.

Although this study provides a set of novel findings, we should acknowledge that the most important question remains unanswered: Why DJ height increased more pronouncedly through the day at HA than at LA? If there was an environmental influence on motoneuron pool excitability it was not detected by our research protocol. Obviously, some additional research needs to be done to clarify whether acute exposure to real moderate altitude affects the explosive or fast stretch-shortening muscle action. To our knowledge, our study was the first attempt to establish a link between the potential neural excitation caused by ascent to real altitude and explosive muscle action, so some limitations of the study were unavoidable. First, because vertical jumps tests could cause a post-activation performance enhancement, which could likely affect the H-reflex measurement, we performed the vertical jump and H-reflex measurements during two separate days. Therefore, the relationship between the vertical jump and H-reflex measurements could be less evident because they were performed on separate days. In addition, the reproducibility of the H/M ratio was lower than for the other variables, therefore, more participants could be needed to detect significant differences in comparisons to the jump-derived variables. Finally, there are big leaps between the neural mechanisms that can be derived from the H reflexes and whether this has an effect on whole-body activity such as drop jumps or squat jumps, so no clear conclusions can be drawn.

## Conclusion

Fifteen min after an ascent to a real moderate altitude the H/M ratio measured at rest increased compared to the morning control values measured at LA. Although this increase might reflect increased excitability of the motoneuron pool, the possible effect of the altitude condition was not confirmed when comparing H/M ratio obtained in LA and HA conditions. In addition, exposure to altitude did not affect the performance of explosive and (SJ) fast stretch shortening (DJ) muscle actions. However, differences in DJ height in favor of HA compared to LA progressively increased throughout the day and reached statistical significance after an altitude exposure of 6 h.

## Data Availability

The raw data supporting the conclusion of this article will be made available by the authors, without undue reservation.
